# Emotional Suppression and Oneiric Expression in Psychosomatic Disorders: Early Manifestations in Emerging Adulthood and Young Patients

**DOI:** 10.3389/fpsyg.2019.01897

**Published:** 2019-08-20

**Authors:** Salvatore Settineri, Fabio Frisone, Angela Alibrandi, Emanuele Maria Merlo

**Affiliations:** ^1^Department of Biomedical and Dental Sciences and Morphofunctional Imaging, University of Messina, Messina, Italy; ^2^Department of Cognitive Sciences, Psychology, Educational and Cultural Studies (COSPECS), University of Messina, Messina, Italy; ^3^CRISCAT (International Research Center for Theoretical and Applied Cognitive Sciences) University of Messina and Universitary Consortium of Eastern Mediterranean, Noto (CUMO), University of Messina, Messina, Italy; ^4^Department of Economics, Unit of Statistical and Mathematical Sciences, University of Messina, Messina, Italy

**Keywords:** psychosomatics, suppression, dreams, psychological factors, emotional

## Abstract

**Background:**

The function of emotions, beginning from the proto-emotions, is the adaptation to the environment. This is based on the *Homeorhesis*, the equilibrium due to the adaptive operation of excitement and the dissipation of emotions. The object relations of the participants foresee the practice of defense mechanisms in a continuum that goes from the consciousness to the oneiric activities. The predominant and maladaptive use of defense mechanisms in the psychosomatic phenomenology, can be identified with deficits in emotional awareness, with the impossibility to manage excitement and dissipation of emotions foreseen by the oneiric phenomena.

**Methods:**

The observation group is composed by 140 participants, 56 males (43%) and 84 females (57%),with pathological-functional disorders of psychosomatic domain. The study had been conducted with the use of measures related to the conscious defense of suppression (Suppression Mental Questionnaire), to the emotional awareness linked to the psychosomatic phenomena (Diagnostic Criteria for Psychosomatic Research Structured Interview-DCPR-SI) and to the states of perturbation and conservation of oneiric activities (The Manheim Dream Questionnaire-MADRE).

**Results:**

Significant inverse correlations emerged among rationalization, repressive function and illness Denial, as for the suppression mental questionnaire factors and irritable mood, but for Regression in the service of the Ego; inverse and significant correlations emerged among suppression, repressive function, rationalization and gastrointestinal psychosomatic outcomes and among suppression, repression and cardiological psychosomatic outcomes. Regarding alexithymia, a positive correlation emerged with rationalization and inverse with Regression in the service of the Ego. Positive correlations emerged between illness denial and overall emotional tone, for disease phobia and meaningfulness and for cardiological psychosomatic outcomes and nightmare distress and recurring nightmares.

**Conclusion:**

The study of such outcomes due to a prevalent defensive style based on suppression, suggest the identification of a key phenomenon, which translates into maladjustment that goes from functional disorders to parasomnia. The bridge established by the obfuscation of conscious contents until the manifestations of disturbance of ancient activities such as oneiric ones, expresses the need to transform an emotional maladaptive style, in line with classic literature and the current state of art.

## Introduction

As suggested by Berney et al. (2014), the clinical study of defense mechanisms allows us to comprehend and to explain affective dynamics, particularly relevant in clinical and therapeutic fields. In line with other defense mechanisms, the suppression has an adaptive meaning when its use is well integrated and directed to the managing of environmental and inner necessities (Metzger, 2014) and it is considered as a mature defense (Perry, 1990; Vaillant, 2000; Perry and Henry, 2004). The use and the polarization on suppression is considered by classical psychosomatic research as the main psychological figure for the occurrence of relevant organic outcomes (Marty and Fain, 1952).

We assist to affective dynamics whose un-expression and difficulties in the mentalization processes, are then translated in body manifestations on target organs and tissues. Differently from other defense mechanisms, the particular constitution of suppressive functioning is based on the need to face conflicts consciously recognized, so that its use is common, conscious and familiar to everyone’s daily life (De Burge, 2001; Settineri et al., 2016).

In this sense, the outcome of the dysfunctional defense use, finding an indirect expression for us known in the products as symbolization, functional disturbance and organic lesions. This particular body-mind dynamic, is close to the current topics of the body-mind problem (Motofei and Rowland, 2018) and the necessity to confront classical approaches to current research practices.

In this sense, some of the classical considerations treated relevant dynamics such as dream contents (Kupper, 1947; French and Shapiro, 1949; Levitan, 1978; Warnes, 1982), dreams preceding psychosomatic illness (Warnes and Finkelstein, 1971) and the failure of defense dynamics in dreams of psychosomatic patients (Levitan, 1981). Recent approaches allow us to maintain the strong basis of previous research (Settineri et al., 2018) with the help of technology and modern practices (Schredl et al., 2014; Settineri et al., 2016, 2019c).

With particular attention to early psychosomatic manifestations and periods preceding somatic crisis, it is known in past literature, how psychosomatic illness is a substitute and following concomitant during emotional states of anxiety, depression, anger, guilt, and fear (Warnes and Finkelstein, 1971; Merlo et al., 2018; Vicario et al., 2019). Dreams are triggered by this emotional state, which in turn facilitates somatic reactivity in predisposed individuals. As for the conscious nature of suppression, the study of dream through consciousness is possible with the empirical research on recall phenomena (Cory et al., 1975; Cernovsky, 1984; Schredl and Engelhardt, 2001; Beaulieu-Prévost and Zadra, 2005; Schredl, 2007; Schredl and Göritz, 2015; Mangiaruga et al., 2018) and with reference to their characteristics and contents (Schredl, 2010). As for the relevance of dream recall, the presence of maladjustment in psychopathological conditions is also expressed through oneiric manifestations (Schredl and Engelhardt, 2001; Llewellyn and Desseilles, 2017; Aviram and Soffer-Dudek, 2018), such as nightmares (Schredl and Göritz, 2018) since childhood to adulthood (Kajeepeta et al., 2015) and above mentioned relevant characteristics of dreaming.

Currently, the need of providing new research instruments close to the purpose of clinical psychology in psychosomatics, was foreseen by the integration of those phenomena that go beyond the nosographic framework (Porcelli and Rafanelli, 2010; Fava et al., 2017). As suggested by Porcelli and Rafanelli (2010), the use of certain psychodiagnostic instruments allow us to diagnose from 2 to 3 syndromes that are not diagnosable with DSM. A particular interest is due to alexithymia, whose relevance has a long history in literature and strong evidence in clinical and neuroscientific contexts (Tesio et al., 2019). The same authors suggest a prevalence psychosomatic syndromes in different clinical settings, such as Dermatology (Picardi et al., 2006), Cardiovascular (Grandi et al., 2001; Rafanelli et al., 2003, 2006), Gastrointestinal (Porcelli et al., 2000; Carrozzino and Porcelli, 2018; Kano et al., 2018), and others.

### The Current Study

The scope of this study was to highlight the characteristics related to the role of different factors of suppression and their outcomes through the revelation of oneiric perturbation and pathological psychosomatic phenomena.

Hypothesis:

HP-1 we hypothesize that different Suppression factors are significantly and inversely (−) associated with the Psychosomatic Syndromes and the pathological diagnosed groups;
HP-2 we hypothesize that the different Psychosomatic Syndromes are significantly and positively associated with Oneiric phenomena, plus the pathological diagnosed groups;

## Materials and Methods

### Procedure and Participants

The observation group consists of 140 participants, 56 males (43%), and 84 females (57%). The age of the participants included in the study is between 19 and 30 years old, with an average of 23.58 years (SD = 2.06).

The research was carried out within the UOC of Psychiatry of the University Hospital G. Martino of Messina.

The participants of the research were considered on the basis of the diagnosed psychosomatic clinical reality, of the related evident psychological components and subsequently subjected to psychodiagnostic practice.

The participants were invited to continue the diagnostic process in order to produce clinical evidence, useful to highlight the psychological characteristics underlying the recognized medical condition and to use the data for research.

The study with the participants and the subsequent diagnostic phases lasted about 2 h for each patient. The compilation of the questionnaires was of a paper and pencil type and each participant before the signing of the informed consent was informed about the anonymous nature of the methods of data processing, as required by the procedures of the ethical committee evidenced by the approval.

### Statistical Analysis

The numerical data were expressed as mean and standard deviation and the categorical variables as number and percentage.

The Spearman test was applied in order to evaluate the correlation among Suppression, Dream and Psychosomatic variables of the following instruments.

Statistical analyses were performed using SPSS 20.0 for Window package.

A *P*-value smaller than 0.050 was considered to be statistically significant.

### Instruments

#### Suppression Mental Questionnaire

The suppression metal questionnaire was developer in 2016 by Settineri et al., it provide for a total score about Suppression and three factors, respectively, Repressive function, Regression in the Service of the Ego and Rationalization. An app version was also developed in 2019 (Settineri et al., 2019c).

The Mannheim Dream Questionnaire was developed by Schredl et al. (2014). An Italian adaptation of the questionnaire was provided by Settineri et al. (2019a). The instrument involves 20 factors about dreams and related phenomena. In this study the selected items were: Overall Emotional Tone, Nightmare Frequency, Nightmare Distress, Recurring Nightmares, Percentage of recurring Nightmares, Meaningfulness.

The reliability indexes of the considered Madre items (Dyck et al., 2017; Settineri et al., 2019a) are reported below.

Overall Emotional Tone:0.764 (0.708 to 0.797)

Nightmare Frequency:0.876 (0.843 to 0.918)

Nightmare Distress:0.823 (0.754 to 0.901)

Recurring Nightmares:0.899 (0.825 to 0.958)

Percentage of recurring Nightmares:0.971 (0.962 to 0.984)

Meaningfulness:0.775 (0.687 to 0.869)

Diagnostic Criteria for Psychosomatic Research (DCPR), is a clinical interview based on diagnostic criteria provided by Fava et al. (1995) whose structured interview is contained in the monograph by Porcelli and Sonino (2007); a set of 12 syndromes was provided: disease phobia, thanatophobia, health anxiety, illness denial, persistent somatization, functional somatic symptoms secondary to a psychiatric disorder, conversion symptoms, anniversary reaction, irritable mood, type A behavior, demoralization, and alexithymia. According to Galeazzi et al. (2004) the items of the DCPR showed a high showed interrater reliability with kappa values:

Disease phobia, kappa 0.97; Thanatophobia, kappa 0.92; Type A behavior, kappa 0.92, Illness denial, kappa 0.90; Demoralization, kappa 0.90; Anniversary reaction, kappa 0.90; Health anxiety, kappa 0.89; Alexithymia, kappa 0.89; Conversion symptoms, kappa 0.82, Persistent somatization, kappa 0.70; Irritable mood, kappa 0.69.

## Results

### Descriptive Statistics

Descriptive statistics (mean and standard deviation) were reported in Tables 1–3, in order to highlight the presence of considered phenomena.

**TABLE 1:** Suppression Mental Questionnaire factors and total score mean and standard deviation.

	**Mean**	**Standard Deviation**
SMQ Total Score	47.78	7.24
Repressive Function	21.20	4.69
Regression in the service of the Ego	16.60	4.16
Rationalization	13.95	2.69
Razionalization and Repressive Function	31.29	5.90
		

**TABLE 2:** Mannheim Dream Questionnaire-Italian Adaptation considered variables mean and standard deviation.

	**Mean**	**Standard Deviation**
Overall Emotional Tone	1.93	0.86
Nightmare Frequency	3.22	2.24
Nightmare Distress	1.56	0.87
Recurring Nightmares	0.38	0.86
Percentage of recurring Nightmares	17.35	17.64
Meaningfulness	1.83	1.00

**TABLE 3:** Diagnostic Criteria for Psychosomatic Research (DCPR) frequency and percentage (*N* = 140).

	**Frequency**	**Percentage**
Health anxiety	123	87.9%
Disease phobia	69	49.3%
Thanatofobia	65	46.4%
Illness denial	62	44.3%
Functional somatic symptoms secondary to a psychiatric disorder	97	69.3%
Persistent somatization	100	71.4%
Conversion symptoms	66	47.1%
Anniversary reaction	57	40.7%
Type A behavior	136	97.1%
Irritable mood	102	72.9%
Demoralization	99	70.7%
Alexithymia	81	57.9%
Dermatological outcomes	117	83.6%
Gastrointestinal outcomes	127	90.7%
Cardiological outcomes	112	80.0%

Hypothesis 1:

As regards the hypothesis 1 and as shown in Table 4, we found significant inverse correlations among Rationalization, Repressive function and Illness Denial, suggesting that decreasing level rationality and suppression corresponds to increasing denial level about diseases consideration and fear.

**TABLE 4:** Correlation coefficients among SMQ and DCPR variables.

	**SMQ Total Score**	**Repressive Function**	**Regression in the service of the Ego**	**Rationalization**	**Rationalization and Repressive Function**
Health anxiety	0.054	–0.028	0.106	0.019	–0.012
Disease phobia	–0.060	–0.110	0.127	–0.087	–0.107
Thanatofobia	–0.021	–0.010	–0.005	–0.049	–0.013
Illness denial	–0.132	–0.136	0.020	–0.162	−0.181^∗^
Functional somatic symptoms secondary to a psychiatric disorder	0.022	–0.012	0.091	–0.062	–0.018
Persistent somatization	–0.085	–0.010	–0.128	–0.056	–0.023
Conversion symptoms	–0.017	–0.030	0.029	–0.035	–0.029
Anniversary reaction	–0.015	–0.030	0.099	–0.045	–0.056
Type A behavior	–0.055	–0.076	0.028	–0.108	–0.091
Irritable mood	−0.198^∗^	–0.257^∗∗^	0.203^∗^	–0.301^∗∗^	–0.302^∗∗^
Demoralization	0.000	–0.091	0.152	0.051	–0.069
Alexithymia	0.014	0.091	−0.185^∗^	0.176^∗^	0.146
Dermatological outcomes	–0.018	–0.057	0.35	–0.067	–0.079
Gastrointestinal outcomes	–0.236^∗∗^	−0.230^∗^	–0.016	–0.123	–0.223^∗∗^
Cardiological outcomes	–0.278^∗∗^	–0.312^∗∗^	0.035	–0.129	–0.284^∗∗^

*^∗^*p* < 0.05 (two-tailed); ^∗∗^*p* < 0.01 (two-tailed).*

Higher scores on Suppression mental questionnaire were associated with lower scores on irritable mood, but for Regression in the service of the Ego. In particular, the increase of Suppression, Repressive function and Rationalization suggest that rational practices related to thought correspond to decreasing irritable mood, anger, and other related phenomena.

Contrary to what emerged about conscious attempts to reduce irritability, the practice of fantasy and inner images as a way to have a deeper understanding of unconscious figures, has an increasing direction as for irritable mood. In this sense, the knowledge about the role of conscious defense such as suppression, confirm the phenomenology of decreasing affective dynamics and mood regulation.

In line with what emerged, Rationalization and Regression in the service of the Ego represent two opposite dynamics. Referring to their relation with Alexithymia, a positive correlation with Rationalization and an inverse relation with Regression in the service of the Ego has emerged. In particular these relations suggest that the non-rationality of the unconscious contents related to emotions are associated with the decreasing of Alexithymia and that a more rational approach to emotions and affectivity is closer to emotive crystallization.

In reference to the use of rationality treating emotions, negative significant correlations emerged among Suppression, Repressive function, Rationalization and Gastrointestinal psychosomatic outcomes. In the same way, negative significant correlations emerged among Suppression, Repression and Cardiological psychosomatic outcomes. In this sense, the directions assumed by the phenomena would inform us about the defensive value of the suppression and the associated phenomena. Specifically, the conscious dynamics linked to pathological outcomes, images and emotions were associated with attempts at adaptation that were still functional for the participants. This is to be considered with respect to the peculiarities of the participants, in their early psychosomatic manifestation. This would represent a good psychotherapic index, designed to avoid a crystallization on certain defense mechanisms (which then turns out to be dysfunctional) and the enrichment of adaptive modalities and management of disease representations.

Hypothesis 2:

As regards the hypothesis 2 and as shown in Table 5, we found that the results involving psychosomatic syndromes and dream outcomes demonstrate positive and significant correlations among Health anxiety with Recurring nightmares, the Percentage of Recurring Nigtmares and Disease Phobia with Meaningfulness.

**TABLE 5:** Correlation coefficients among DCPR and Madre variables.

	***Overall Emotional Tone***	***Nightmare Frequency***	**Nightmare Distress**	**Recurring Nightmares**	**Percentage of recurring Nightmares**	**Meaningfulness**
Health anxiety	–0.120	–0.008	0.055	–0.017	0.177^∗^	0.026
Disease phobia	–0.002	0.136	0.102	0.136	–0.023	0.216^∗^
Thanatofobia	−0.207^∗^	–0.060	–0.009	0.045	0.076	0.074
Illness denial	0.180^∗^	0.107	0.122	–0.107	0.066	0.019
Functional somatic symptoms secondary to a psychiatric disorder	0.054	0.024	0.068	0.029	–0.017	0.052
Persistent somatization	0.139	–0.015	–0.086	0.008	0.001	0.036
Conversion symptoms	0.004	0.132	0.001	0.095	0.045	0.004
Anniversary reaction	0.092	−0.200^∗^	0.028	0.035	–0.029	0.059
Type A behavior	0.023	0.078	0.069	–0.061	–0.001	0.095
Irritable mood	–0.071	–0.092	0.088	0.076	–0.003	0.122
Demoralization	–0.122	0.149	–0.026	–0.026	–0.014	–0.123
Alexithymia	–0.160	0.002	–0.057	–0.057	0.059	–0.088
Dermatological outcomes	–0.114	0.017	0.038	0.038	–0.059	–0.049
Gastrointestinal outcomes	–0.049	0.079	0.114	0.114	–0.006	0.013
Cardiological outcomes	0.061	0.166	0.177^∗^	0.177^∗^	–0.098	0.069

*^∗^*p* < 0.05 (two-tailed); ^∗∗^*p* < 0.01 (two-tailed).*

In particular, considering the participants involved in this study with their early psychosomatic manifestations, these results show that at the beginning of the somatic manifestations we assist to an association linked to health and corporeal anxiety and the oneiric disturbance.

Beyond the highlighted associations, it is known as the managing of excitement and the dissipation of emotions ceases to be adaptive in pathological experience.

In the same direction, Illness Denial and Overall emotional tone were in a positive relation, suggesting the direction of emotions involved in this interruption of the adaptive managing of emotions. As for other relevant phenomena, the meaning related to dreams and to experience have a strong role in the adaptation processes. In particular, a positive correlation emerged between Disease Phobia and Meaningfulness, where the increase of phobias related to health and diseases corresponds to the increase of search for meaning.

In line with oneiric disturbance, Cardiological psychosomatic outcomes had positive associations with Nigtmare Distress and Recurring Nightmares, in order to confirm the relevance of dreaming related to the perception of somatic anguish and agitation.

## Discussion

The current study was aimed at highlighting the relationships between emotional suppression and typical oneiric expressions in psychosomatic individuals in their first pathological manifestations.

For this reason, the choice to use specific instruments for the phenomena expressed, was been extended to participants who experimented pathological experiences of dermatological, cardiological and gastroenterological domain. The results showed that it was possible to observe phenomena related to emotional suppression in association with psychosomatic dynamics and between dream expressions and disorders mentioned above. In support of the first hypothesis of this study, we found an inverse general trend was found regarding the practice of emotional suppression, in relation to specific psychosomatic realities and large diagnostic groups. Considering the different nature between the first two factors of repression and the regression in the service of the Ego (less conscious fantasies), an important datum emerged in relation to the continuous and positive trend of the aforementioned dynamic and psychosomatic factors. In our research experience and in line with what was expressed in the results, conscious attempts to stem the psychosomatic experience appear in a reverse and therefore functional direction. In support of the second hypothesis, regarding oneiric manifestations we found dysfunctional experiences emerged in positive association with psychosomatic pathological experiences. Nightmares and dysfunctional emotionality emerged positively associated with specific psychosomatic dynamics and in particular (recurrence of nightmares) with cardiac disorders.

It is known how hard the impossibility to distance ourselves from pathological phenomena can be, such as psychosomatic ones, since childhood (Szwec, 2018). Our experience was referred to participants in their early pathological manifestations and to the reasons for symptom formation, all conflicts, defenses and previous traumatic experiences (Wolf et al., 2018). The main scope of this research was to highlight directly and undirectly related phenomena, in order to prevent the typical loss of an adaptive Self (Marchini et al., 2018) and psychological manifestations whose mechanisms can predict the serious incoming pathological realities, their complex denial (Livneh, 2009) and subsequent decreasing quality of life and body satisfaction (Guicciardi et al., 2014; Catalano et al., 2018; Martino et al., 2018) for both patients and caregivers (Ferrario et al., 2017; Settineri et al., 2019b).

In our experience, rationality and conscious suppressive functions emerged as opposite to denial phenomena, so that the defense function appears as an adaptive way to manage emotions related to the pathological reality. This result was in line with recent perspectives about denial, where the managing of negative emotions due to the onset of pathological manifestations, together with resistance to change, represent a relevant step in the conscious elaboration of disease (Nowak et al., 2015; Ferrario et al., 2017).

As suggested by Ferrario et al. (2017) the unconscious nature of defense mechanisms, different to coping strategies, is unintentionally (Cramer, 2000). Our case involved a conscious phenomenon, so the inverse relation between suppression and denial confirm the distance and the effect that each factor had on the other.

As for the unconscious nature of denial, the reasons on the basis of mood misadjustment can be unknown. The role of suppression on negative emotions, such those related to irritability and anger, was found as relevant in our experience. As suggested by Wolf et al. (2018), the onset of somatic symptoms involves different defense mechanisms, useful to manage emotional distress.

In particular neurotic manifestations such as irritability, tension, anxiety, impulse have the role to avoid intolerable feelings. The role of the studied factors in our contributions, distinguishes between defenses closer to consciousness and others, related to unconscious necessities. In line with the emerged results in the case of denial, even for irritable mood, the role of rational functions has an effective decreasing role, contrary to regression dynamics. In our experience, the role of fantasy and rationality changes in front of alexithymia and his relation with physical illness (Porcelli and Taylor, 2018).

According to Porcelli and Taylor (2018), the role of alexithymia in the production of somatic symptoms is relevant. As for the inverse effect of suppression on alexithymia (and the positive effect of fantasy and regression in the service of the Ego), the results highlighted the same dynamic also extended to gastrointestinal and cardiovascular disorders, according to Porcelli and Taylor and the above-mentioned literature about somatic symptoms and research methodology.

With particular reference to those pathological psychosomatic outcomes, other relationships emerged in term of oneiric manifestations. Health anxiety and illness denial were directly related to nightmares, in terms of recurring phenomena and emotional tone. The link between nightmares distress and frequency with psychosomatic manifestations is known in literature (Nguyen et al., 2002; Fantoni et al., 2007; Molina et al., 2016).

In our experience the central emotive core of fear about diseases, involved the search of sense and meaning, as expressed by dreams (Levitan, 1976), as highlighted by the relation between the conscious consideration of anniversaries and the decreasing manifestation of nightmares. In line with the above-mentioned considerations about the innovative use of instruments related to psychosomatics, we suggest our research experience, as linked to the integration and promotion of psychodynamic phenomena, before less considered clinically and psychometrically. Our direct experience with the construction of a psychometric instrument linked to suppression is close to this purpose. The use of this instrument represented a clinical consideration specifically dedicated to the primary phenomena associated with suppression and consequently to psychosomatics.

### Limitations and Conclusion

This study has a diversity of limitations that should be overcome in future studies. Besides the small sample size (140 participants), we assist just to the early psychosomatic manifestations in our young patients. This fact can be read through two opposing points of view. The first concerns this precise study, in which the first pathological manifestations are still not chronic and structured, although there are significant physical outcomes. This state is justified by the difficulty to express disturbing contents, which justifies the use of projective methods. Through the use of pareidolia and apperception (respectively, for Rorschach and Thematic Apperception Test) we reach equivalents whose revelation is due to the indirect request. The use of scales and psychometric measures is always useful, more with large samples and advanced pathological realities. The second point of view concerns the occasion given by this kind of studies, in terms of psychotherapy interventions. More advanced pathological states give light to a large number of relations among different variables, even if it could be harder to intervene on the phenomena in progress. Our purpose is referred to young patients, and in particular to the possibility to notice those phenomena on which it is possible to intervene instantly.

Further studies should focus in particular on investigating the relationships emerged, and should explore the causal links for which the outcomes we are witnessing are structured.

## Data Availability

The datasets generated for this study are available on request to the corresponding author.

## Ethics Statement

This study was approved by the local Institutional Ethical Committee. All the participants gave their consent to participate in this study and were evaluated by the clinical psychologists and physicians. This research was conducted with respect for the rights of the participants, according to the World Medical Association Declaration of Helsinki and its amendments. All administered instruments, including questionnaires, rating scales, and clinical structured interviews, were performed as a part of normal clinical practice assessment of patients. The data was analyzed anonymously. Each participant was properly informed about the research aim and study, and after comprehension signed the informed written consent.

## Author Contributions

All authors listed have made a substantial, direct and intellectual contribution to the work, and approved it for publication. SS made significant contribution to design the research study, and draft the manuscript and revise it critically. FF provided substantial contribution in drafting the part of the manuscript. AA performed the statistical analysis and provided significant contribution to draft the part of the manuscript. EM made significant contribution to design the research study, provided the interpretation of data, a substantial contribution to draft the manuscript, and gave the final approval.

## Conflict of Interest Statement

The authors declare that the research was conducted in the absence of any commercial or financial relationships that could be construed as a potential conflict of interest.
